# Supplementary material for: Strain variation and anomalous climate synergistically influence cholera pandemics
Source: PLoS Negl Trop Dis. 2024 Aug 1;18(8):e0012275. doi: 10.1371/journal.pntd.0012275 (PMC11293675; doi:10.1371/journal.pntd.0012275)
Supplement: S1 Supplementary Information — (DOCX) [file pntd.0012275.s001.docx]

**Supplementary Information**

**Datasets:**

Historical cholera incidence datasets were retrieved from the annual reports of the Sanitary Commissioner for 1900 and 1901 in Bengal^20^ and Punjab^21^ and in Calcutta^22^)

In order to also cover the recent period, the gridded rainfall data from Global Precipitation Climatology Project (GPCC)^25^ were used for Bangladesh (retrieved from <ftp://ftp.dwd.de/pub/data/gpcc/html/fulldata_v6_doi_download.html>). These data are available at a 0.5^o^ resolution for the whole 20^th^ century (1901-2012), and have undergone a strict homogenisation and quality control procedure to remove abnormal outliers and station biases. To assess whether the extended dataset in the Global Precipitation Climatology Project (GPCC^25^) can replace station rainfall for larger spatial domains than those of single stations, correlations were calculated between the first two PCs of the rainfall station data and those of the GPCC reanalysis database. Correlations are both significant (r_PC1_= 0.79 and r_PC2_= -0.67, respectively, *p<0.005*), indicating that the GPCC dataset is here a sufficiently good analogue of station rainfall. The Twentieth Century Reanalysis (TCR) dataset for the region was also analysed for variables that can help better track climatic influence. Among these, sea-surface temperatures (SST) serve to better visualize weather dynamics related to local rainfall.

The Niño3.4 SST index of ENSO was considered to address associations with cholera (<http://www.esrl.noaa.gov/psd/people/cathy.smith/best/>). Niño 3.4 represents the sea surface temperature anomalies over a small region of the tropical Pacific near the coast of South America.

**Methods**

Multi-channel singular spectrum analysis (MCSSA)^31,32^ is a multivariate version of the singular spectrum analysis (SSA) method, a nonparametric technique for the decomposition of time series into major orthogonal components. In SSA, these components are the eigenvectors of a covariance matrix constructed from a given times series against itself at different lags (for a total number of prescribed lags). The amount of variance corresponding to the specific components (including cyclical ones which come in pairs) is indicated by the corresponding eigenvalues of the eigenvectors. Components can correspond to trends, cycles (of different frequencies, including seasonality and interannual variability) and noise. Once the major components are identified, a reconstruction of the temporal pattern of variation can be obtained from a selected set. Thus, the reconstructed time series allow selection of a range (or a specific) time scale, as well as the removal of noise and trends.

# We used the SDC analysis^30, 45^ to study the local and transient patterns of variability, in particular the local correlations existent between different variables. Specifically, the two-way SDC (TW-SDC) technique computes non-parametric Spearman rank correlations between two time series in localized windows of time, using rcorr (<https://www.rdocumentation.org/packages/Hmisc/versions/4.2-0/topics/rcorr>). TW-SDC are computed at a variety of window sizes, S, and at different lags between the two variables . A correlation value is obtained at a given significance level (p< 0.05 or 0.01, as indicated). The SDC method is optimal for correlations between short and noisy time series, and because it is sensitive to window size, different S values, above and below the period of interest, are applied to assess for consistency in the results.

We carried out a continuous wavelet transform (CWT) procedure, using Morlet wavelets as the basis function, to generate the cross-wavelet power plots in Fig 2A. Morlet wavelets are localized in both time and frequency (= 1/period) domain, and CWT has the capability to provide a continuous resolution along the period axis. These two features together have enabled us to isolate the well-defined seasonal synchrony patterns in the year 1905 in these plots.

A Morlet wavelet along the time axis *t* is a plane wave modulated by a Gaussian^1^:

$\psi(t)=\pi^{{-1}/4}e^{i\omega t}e^{{-t^{2}}/2}$, (1)

where $e^{i\omega t}$ is the plane wave function with (angular) frequency $\omega$ and $e^{{-t^{2}}/2}$ is the Gaussian envelope that exponentially dampens the waveform to achieve localization. CWT of a discrete time series $x_{n}(n=1...N)$ is a convolution of $x_{n}$ with a set of conjugate Morlet instances $\psi^{\text{*}}(t)$, translated along *localized* time points *m* and scaled by the *period* *s*:

$W_{m}(s)=\sum_{n=0}^{N-1} x_{n}\psi^{\text{*}}[\frac{(m-n)\Delta t}{s}]$, (2)

where ∆*t* is one time step that relates the discrete time index *n* to continuous time *t*. To speed up computation, the convolution (2) is implemented as an inverse Fourier transform:

$W_{m}(s)=\sum_{k=0}^{N-1} \hat{x_{k}}\hat{\psi}^{\text{*}}(s\omega_{k})e^{i\omega_{k}m\Delta t}$, (3)

where $\hat{x_{k}}(k=1...N)$ is the discrete Fourier transform (DFT) of $x_{n}$ and likewise $\hat{\psi}^{\text{*}}(s\omega_{k})$ is the DFT of $\psi^{\text{*}}(t/s)$, *k* being the Fourier frequency index (not the wavelet frequency).

For two time series $x_{n}$ and $y_{n}$ and their respective CWTs $W_{n}^{x}(s)$ and $W_{n}^{y}(s)$, the cross-wavelet spectrum is given by $W_{n}^{xy}(s)=W_{n}^{x}(s)W_{n}^{y\text{*}}(s)$, where $W_{n}^{y\text{*}}$ is the complex conjugate of $W_{n}^{y}$. $W_{n}^{xy}(s)$ is a complex object, hence its modulus $|W_{n}^{xy}(s)|$ is defined as the *cross-wavelet power*.

The cholera mortality data includes both seasonal and higher-scale signals of interest as well as high-frequency noise. In order to extract unambiguous information, we first de-noised each dataset by removing all cycles below the 8-month period. After de-noising, we computed the cross-wavelet power $|W_{n}^{xy}(s)|$ of each data pair $(x_{n},y_{n})$ to generate the plots in Fig2A.

The WaveletComp1.1 package^2^ in R^3^ was used for our analysis.

# References:

# 1. Torrence C. and Compo G.P. (1998). A practical guide to wavelet analysis. Bulletin of the American Meteorological Society 79 (1), 61–78.

# 2. Roesch A. and Schmidbauer H. (2018). WaveletComp: Computational Wavelet Analysis. R package version 1.1. <https://CRAN.R-project.org/package=WaveletComp>.

# 3. R Core Team (2024). R: A Language and Environment for Statistical Computing. R Foundation for Statistical Computing, Vienna, Austria. <https://www.R-project.org/>.
