# Supplementary figures and images for: Strain variation and anomalous climate synergistically influence cholera pandemics
Source: PLoS Negl Trop Dis. 2024 Aug 1;18(8):e0012275. doi: 10.1371/journal.pntd.0012275 (PMC11293675; doi:10.1371/journal.pntd.0012275)

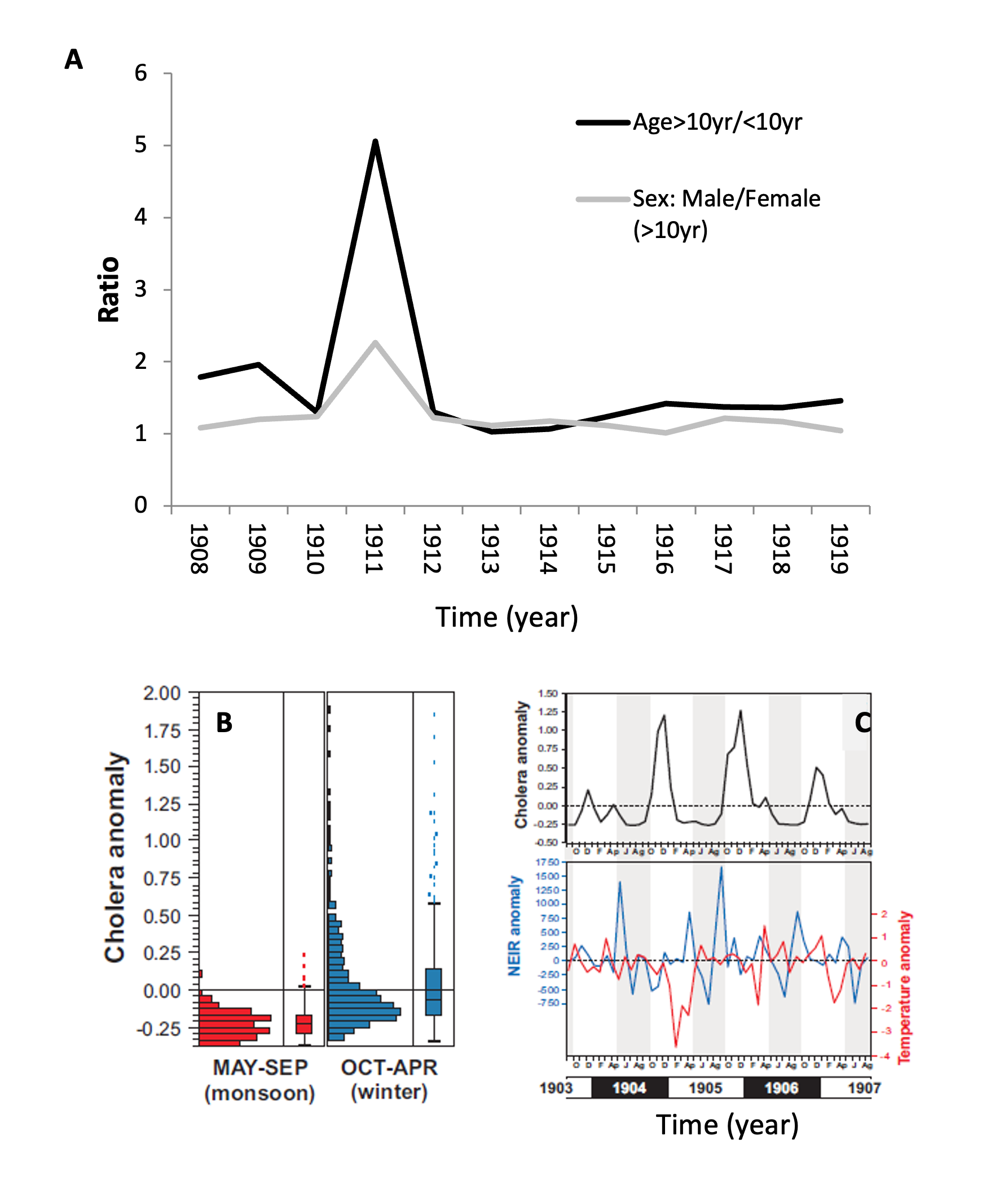

Supplement: S1 Fig — Relationships between rainfall (10−1mm/month) and temperature (deg. °C/day) in Dacca for the reference period of 1893–1935, together with anomalies in cholera mortality: A) distribution of monthly rainfall (NEIR) and temperatures, with means indicated by large circles (J—January, F—February, Mr—March, Ap—April, Au—August, S—September, O—October, N—November, and D—December); B) distribution of total occurrences of cholera mortality anomalies during the monsoons (May to September) and in winter (October to April); C) evolution of cholera mortality, rainfall and temperature during the anomalous 1904–07 event (grey background stripes correspond to the monsoon period). (TIF) [file pntd.0012275.s002.tif]

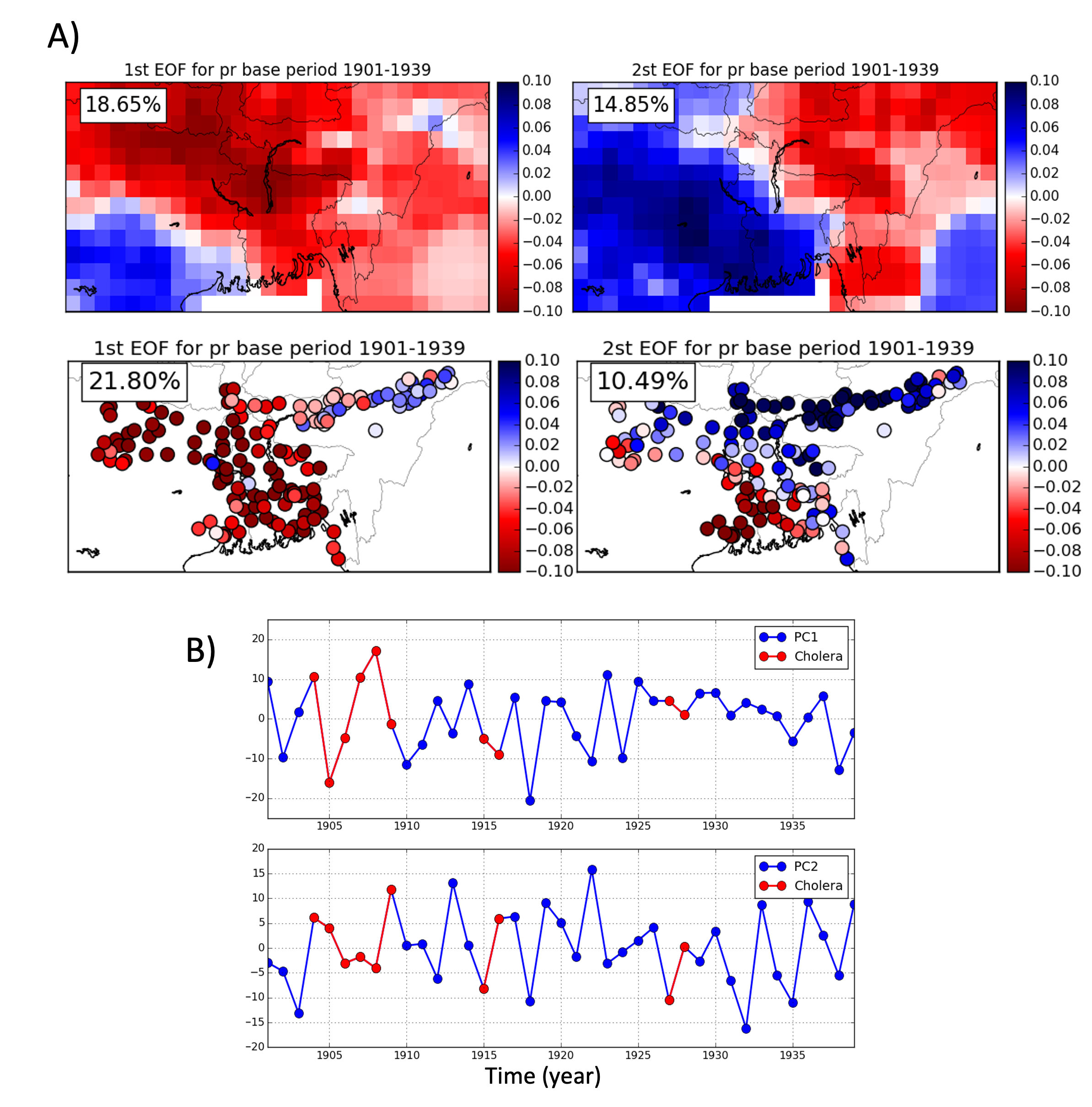

Supplement: S2 Fig — A) Principal Component Analysis (PCA) of the de-seasonalized GPCC rainfall reanalysis [25] over Bangladesh for the interval 1901–1940. Years before 1901 were not available. Empirical Orthogonal Functions (EOF) are significant at the p<0.01 level. Bottom row depicts the results of a similar PCA but applied on ground station data. B) Temporal PCs (t-PC) for the EOF components in A) with red years denoting large cholera anomalies in Bengal (note that the sign is arbitrary in PCA). (TIF) [file pntd.0012275.s003.tif]
