# Supplementary material for: Strain variation and anomalous climate synergistically influence cholera pandemics
Source: PLoS Negl Trop Dis. 2024 Aug 1;18(8):e0012275. doi: 10.1371/journal.pntd.0012275 (PMC11293675; doi:10.1371/journal.pntd.0012275)
Supplement: Attachment — Submitted filename: PLoS NTD_Resposes to Reviewers.pdf [file pntd.0012275.s005.pdf]

Responses to Reviewers' comments

(Our responses are in bold after each comment)

Reviewer #1:
The authors examine the role of climate (temperature, precipitation, and El Niño) in the cholera pandemic of 1899-1923) in former British India (Bengal, or present-day Bangladesh), and generate climate-based projections. The authors conclude that rainfall anomalies toward the beginning of the outbreak were extreme (statistically) and played a role, and that increasing variability and frequency of such extremes is of concern for future pandemics.
For context, I'm a physical climate scientist with relatively little experience with public health, epidemiology, etc. I've collaborated on some climate/health studies, but my contributions have largely been in the climate sphere (observations and global climate modeling). My comments mostly focus on those aspects of this study.
My overall impression is that this is a nice study worthy of publication in PLOS NTD after some revisions. It's clear to me that the authors are disease folks who are doing their best with climate observations and models. I mean that in a nice way. I give the authors an A on the novelty and probably everything else, but a B at best with the climate analysis, and I don't think they're going to get to an A without collaboration with a climate specialist. To be blunt, most of the climate stuff here would be torn apart in a climate journal, but that's not the point of interdisciplinary studies like this in PLOS NTD. What's important is that I don't think anything with the climate aspect of this study is concerning enough to me that it undermines the overall conclusions. I actually buy the conclusions.

R: We appreciate the positive remarks by the reviewer. We clarified the climate analysis, to further explain the relevance and significance of the climate results and how they strengthen the proposed relationship with cholera outbreaks/pandemics. We agree that these results do not constitute a complex climate analysis, but we believe that despite their simplicity, their robustness and clarity provide solid evidence for the proposed relationship between climate anomalies and cholera outbreaks/strain replacement. As the referee states the overall conclusions are clearly aligned with the results presented in the study.

INTRODUCTION

I enjoyed the introduction. I found it easy to follow and well referenced. Just one minor thing—on page 5, "interaction" is probably not the right word, unless you're suggesting the pandemic feeds back onto climate.

R: Agreed, thank you for highlighting the potentially confusing text. We edited this text as follows: "the role of climate conditions on strain variation".

DATA AND METHODS

The cholera dataset seems like an amazing resource. The data are organized monthly by villages, so the spatiotemporal sampling and resolution seems appropriate for the analysis objectives. Reliable climate data from this period (turn of the 20th century) are challenging to

come by, but due in part to the colonial enterprise, relatively good records from this region were kept at the time. I have a few comments about the climate data.

R: Thank you very much for the comments.

The datasets used seem appropriate, but note that SST is not really "from" the 20th Century Reanalysis. That is an atmospheric reanalysis/data assimilation product and SSTs are prescribed from another source. That sources is called SODA. Please review the information on this page under the Description tab: https://www.psl.noaa.gov/data/gridded/data.20thC_ReanV3.html

Which version of the NOAA 20th Century Reanalysis is used? There are three at this point.

R: Thank you for noticing this problem. Our study underwent a series of updates in time and as a result, some errors were introduced in the text. One was the accidental removal of the source information for the SST dataset, which as the reviewer correctly points out, is not the NOAA 20th Century Reanalysis. We have now added the correct source to refer to the Kaplan Extended SST V2 dataset, which includes gridded SST anomalies from 1856 to the present, with respect to a climatology spanning the period of 1951-1980. It is based on the UK Met Office SST data and on various statistical techniques applied to fill in missing data. For more details, we refer to Kaplan et al. (1998) and Reynolds and Smith (1994). We have updated this information on lines 142-146.

You refer to the Global Precipitation Climatology Product, but then use the acronym GPCC. The problem is that there actually are two different precip datasets: GPCP and GPCC. I believe you are using GPCC but please correct the acronym to eliminate the ambiguity.

R: Agreed, it was a misunderstanding and it has now been corrected to read GPCC (lines 288,296). We used version GPCC-FD from the Global Precipitation Climatology Centre database
(doi:10.5676/DWD GPCC/FD M V6 050, doi:10.5676/DWD GPCC/FD M V6 100) as stated in Becker et al., Earth Syst. Sci. Data, 5, 71–99 (2013), and as such it had been cited already in the former version of the manuscript.

The statistical techniques seem standard and appropriate, including the use of EOF analysis with the climate data.

R: Thank you for the positive assessment.

I have an issue with the climate models, though. The scientific community has had free and open access to CMIP6 for many years now. Without a specific justification for using CMIP5 rather than CMIP6, I cannot say I approve of this. … However, I note that in the SI (and only in the SI), it is stated that CMIP5 and CMIP6 models are used. This needs to be harmonized with what is presented in the main text. I later see that Fig. 5 includes both generations of models, and both are discussed in the Discussion and conclusions section. I guess the methods section in the main text just needs to be updated to indicate that CMIP6 models are also included.

R: We thank the reviewer for pointing out this error. As stated before, the manuscript has undergone a series of rewrites over time, and the information has not been properly updated. As the reviewer correctly points out, in Figure 6 (previous Fig. 5) we have used models from both the CMIP5 and CMIP6 generations due to the differences that have been generated in the area of former Bengal in the latter with regard to the former. We have further discussed these discrepancies (lines 465-484) and also updated the information in the Methods section on lines 189-206 and the Results section (lines 345-363) to reflect this.

The authors say that one thousand multi-model simulations based on CMIP5 were averaged. I'd like to understand where the number 1,000 comes from. According to the SI, the authors used 30 models from CMIP5 and 30 models from CMIP6. That implies an average of 16.7 ensemble members from each model are being used. I do not see discussion of ensemble members, so it's not clear where this is coming from. From the description in SI, it almost sounds like the authors mean members of a composite (compositing on ENSO phases), which is not the same as ensemble runs (where the initial conditions are varied) from climate models.

R: We agree that t**his information led to confusion and we have rewritten it for further clarity. For CMIP5 and CMIP6, we explain that we have used only one ensemble member from each model. The previous mention was to a previous figure that is no longer included. We updated the information regarding the use of CMIP5 and CMIP6 models for the preparation of Figure 6 and on lines starting at 348.**

RESULTS

It's not clear what is plotted in Figure 3A. The caption does not help. Difference between the average of the 1904-08 event and the seasonal cycles of … over what time period? What is being differenced, exactly?

R: The difference mentioned is for the variable of interest (precipitation, temperature), between the average of each month for the interval 1904-08 and the average of that same month in the interval 1893-1939, excluding the 1904-08 period. This sentence has been rewritten for clarity in the caption of Fig. 3 and in the text, as follows: "Fig. 3A summarizes the anomalous seasonal patterns of mortality, rainfall and temperature respectively for Dacca during the exceptional period of 1904-07. The graphs show the difference between the average value for each given month over this period and the average for the same month for the period of 1893-1939)."

What do the authors mean by a 21 degree temperature drop? I don't see anything like that on any of the figures. Is the red line on 3A scaled by 0.1 or something?

R: Thank you for noticing this typo in the writing. As the reviewer can see in the associated figures, the value should be 2.1 degC as now corrected in line 267. Fig. 3C properly depicts the change.

Are the EOF results shown in S2 conducted on annual mean precip, seasonal mean precip, or monthly? If monthly, I really hope the authors are removing the climatological seasonal

cycle first, otherwise the first mode will of course be the annual cycle. These details need to be provided in the methods section(s), if not the captions as well.

R: Yes, seasonality was of course removed prior to this analysis. We have now indicated this step in the text (l. 289), and in Fig. S2 caption.

Please don't show the 3rd and 4th mode. They are obviously statistical noise and are (thankfully) not analyzed.

R: Thank you. We did not use those two modes and they have now been removed from the figure.

Page 11: Just a friendly writing suggestion: sentences like "highly anomalous cholera years are indicated as red dots" belong in captions, not in main text.

R: Agreed, we removed this sentence from the main text, as it was already specified in the caption of Fig. S2.

The global correlation maps between SST and NINO3.4 (Fig. S3A) are kind of bizarre, I have to say. There's nothing new there, and it's not clear how those connect to cholera (S3B) and S3B is extremely hard to read. I cannot make out the years on the x-axis. It's possible there is something really important here, especially with the supposed result that cholera lags ENSO by 7-12 months, but I definitely don't see it from reading the top paragraph on page 13 and looking at Fig. S3.

R: Thank you for this comment. In taking into account how to best address this comment, we also considered a related but different comment on this same figure by the other reviewer, who highlighted the relevance of the different oceanic SSTa configurations for each EN event. The other referee specifically stressed the relevance of this figure and asked that it be included in the main manuscript. We therefore opted for improving this figure and making clearer the specific contribution it makes. Results in the new Fig. 5 not only illustrate the different delays between the effector driver and the response in cholera, but they also show how different strong EN events have had different signatures in terms of their SSTa, both in the tropical Pacific and in the northern Indian Ocean, close to Bangladesh. By highlighting such different patterns of regional ocean warming, consistent with other published studies, we seek to indicate that such differential ocean warming might lead to different rainfall modulations in the

region of interest, ultimately affecting the size and timing of ensuing cholera events.

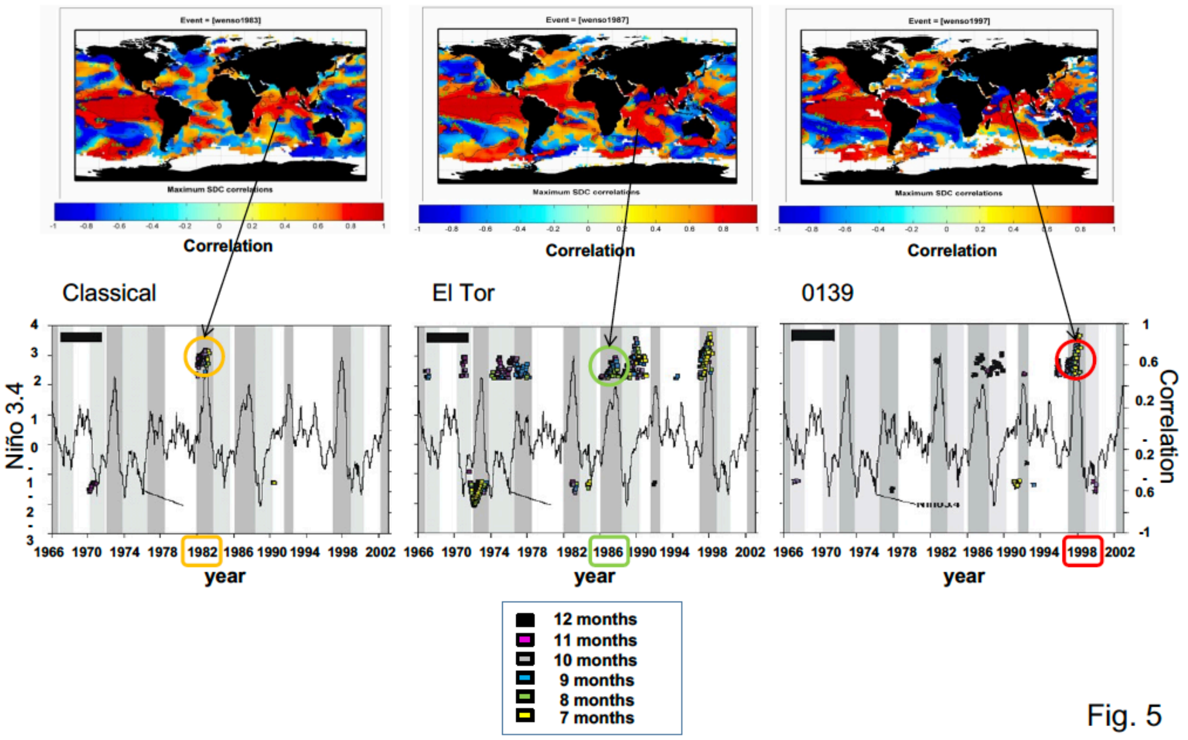

Fig. 5

The actual role for climate models (CMIP5/6) in this paper is very minor. It boils down to a pretty poorly done figure tacked on at the end of the paper (Fig. 5). The color scales in the maps are extremely saturated and no indication of statistical significance is given. Also, annual? Seasonal? All months? Note that if annual, it's not a good idea to composite annual mean fields onto ENSO since ENSO peaks at the breakpoint between calendar years.

R: The description of the methodology on which Figure 6 is based is now given in the main text on lines 189-206. The ENSO composites are shown for the December-January-February period, the usual time when ENSO events peak. We have also improved panels A and B in Figure 6. The results shown in Figure 6 are now summarized on lines 345-363 and discussed (in the Discussion section) on lines 465-484.

DISCUSSION AND CONCLUSIONS

Nice summary and connection to climate literature about the future of ENSO. No concerns here. I don't detect exaggeration of the results or their significance.

R: Thank you again for your positive feedback.

Reviewer #2: (No Response)

PLOS authors have the option to publish the peer review history of their article (<a href="https://journals.plos.org/plosntds/s/editorial-and-peer-review-process#loc-peer-review-

history" target="_blank">what does this mean?). If published, this will include your full peer review and any attached files.

If you choose “no”, your identity will remain anonymous but your review may still be made public.

Do you want your identity to be public for this peer review? For information about this choice, including consent withdrawal, please see our Privacy Policy.

Reviewer #1: No

Reviewer #2: No

Figure Files:
While revising your submission, please upload your figure files to the Preflight Analysis and Conversion Engine (PACE) digital diagnostic tool, https://pacev2.apexcovantage.com. PACE helps ensure that figures meet PLOS requirements. To use PACE, you must first register as a user. Then, login and navigate to the UPLOAD tab, where you will find detailed instructions on how to use the tool. If you encounter any issues or have any questions when using PACE, please email us at figures@plos.org.
R: We have checked all figures with the PACE tool.

Data Requirements:
Please note that, as a condition of publication, PLOS' data policy requires that you make available all data used to draw the conclusions outlined in your manuscript. Data must be deposited in an appropriate repository, included within the body of the manuscript, or uploaded as supporting information. This includes all numerical values that were used to generate graphs, histograms etc.. For an example see here: http://www.plosbiology.org/article/info%3Adoi%2F10.1371%2Fjournal.pbio.1001908#s5.
R: We have provided proper reference and link to the open access of the relevant data included in this study.

Reproducibility:
To enhance the reproducibility of your results, we recommend that you deposit your laboratory protocols in protocols.io, where a protocol can be assigned its own identifier (DOI) such that it can be cited independently in the future. Additionally, PLOS ONE offers an option to publish peer-reviewed clinical study protocols. Read more information on sharing protocols at https://plos.org/protocols?utm_medium=editorial-email&utm_source=authorletters&utm_campaign=protocols

References
Please review your reference list to ensure that it is complete and correct. If you have cited papers that have been retracted, please include the rationale for doing so in the manuscript text, or remove these references and replace them with relevant current references. Any

changes to the reference list should be mentioned in the rebuttal letter that accompanies your revised manuscript. If you need to cite a retracted article, indicate the article's retracted status in the References list and also include a citation and full reference for the retraction notice.

Attached file

In the manuscript (ID: PNTD-D-24-00277) entitled ‘Strain variation and anomalous climate synergistically influence cholera pandemics’ authors investigated the role of climate variability in the emergence of pathogenic cholera variants. Here, this synergy was established by analysing extensive data from historical records of climate variables and cholera mortality at the time of 6 th and 7 th cholera pandemics in provinces of former British India. The team has also used the historical data for future climate projection with the help of multi model climate simulations. The study is well designed and nicely executed. The manuscript is written well with a logical flow of information and a perceptive discussion. This work will certainly add value to the existing literature of cholera episodes with their major external drivers.

R: Thank you very much for the positive evaluation of our study.

However, this reviewer has some suggestions.

1. Graph representing correlation between weather anomalies (in different time period and places) and corresponding emergent cholera strains (Classical/El Tor/ O139) should be included in the manuscript. In this context, supplementary figure 3 should be moved to main manuscript figures.

R: Thank you for the suggestion. We now rearranged this figure for clarity and moved former supplementary figure 3 to the main text as Fig. 5.

2. Future climate projection (Figure 5) was established using CMIP5 and CMIP6. However, the importance of this findings in predicting possible cholera episodes (with possible responsible strains) was not clearly discussed.

R: Thank you for this comment. We have now extended the description of Figure 6 (previously Fig. 5) in lines 345-363. We also specifically discuss the importance of these findings for the possible emergence of cholera strain changes in the future on lines 465-484.

3. ‘Results’ will be more clarified and understandable if written under different headings/ subheadings depicting major findings. Same goes for ‘Data and methods’.

R: Thank you. We have proceeded to include subtitles/subheadings in the different sections.

4. Limitation of the study and scope for future work should be discussed in the manuscript.
R: Agreed. We have added a paragraph at the end of the Discussion.

5. Axis legends and keys of figure 4 and 5 are difficult to read.
R: Agreed. All figures and legends are checked for readability.

6. In the caption of figure 3, numbering of figures do not match with the graphs (should be C1 and C2 instead of B3 and B4).
R: Thank you. For simplification and clarity, this notation has been replaced by A-B-C.

7. Expand the abbreviation JJAS mentioned in figure 3.
R: Thank you, corrected accordingly.
