# Supplementary material for: Strain variation and anomalous climate synergistically influence cholera pandemics
Source: PLoS Negl Trop Dis. 2024 Aug 1;18(8):e0012275. doi: 10.1371/journal.pntd.0012275 (PMC11293675; doi:10.1371/journal.pntd.0012275)
Supplement: Attachment — Submitted filename: Comments_PNTD-D-24-00277.docx [file pntd.0012275.s004.docx]

In the manuscript (ID: PNTD-D-24-00277) entitled ‘Strain variation and anomalous climate synergistically influence cholera pandemics ’ authors investigated the role of climate variability in the emergence of pathogenic cholera variants. Here, this synergy was established by analysing extensive data from historical records of climate variables and cholera mortality at the time of 6^th^ and 7^th^ cholera pandemics in provinces of former British India. The team has also used the historical data for future climate projection with the help of multi model climate simulations. The study is well designed and nicely executed. The manuscript is written well with a logical flow of information and a perceptive discussion. This work will certainly add value to the existing literature of cholera episodes with their major external drivers.

However, this reviewer has some suggestions.

1. Graph representing correlation between weather anomalies (in different time period and places) and corresponding emergent cholera strains (Classical/El Tor/ O139) should be included in the manuscript. In this context, supplementary figure 3 should be moved to main manuscript figures.
2. Future climate projection (Figure 5) was established using CMIP5 and CMIP6. However, the importance of this findings in predicting possible cholera episodes (with possible responsible strains) was not clearly discussed.
3. ‘Results’ will be more clarified and understandable if written under different headings/ subheadings depicting major findings. Same goes for ‘Data and methods’.
4. Limitation of the study and scope for future work should be discussed in the manuscript.
5. Axis legends and keys of figure 4 and 5 are difficult to read.
6. In the caption of figure 3, numbering of figures do not match with the graphs ( should be C1 and C2 instead of B3 and B4).
7. Expand the abbreviation JJAS mentioned in figure 3.
